# COVID-19: room for treating T cell exhaustion?

**DOI:** 10.1186/s13054-020-02960-0

**Published:** 2020-05-15

**Authors:** Giovanni Riva, Vincenzo Nasillo, Enrico Tagliafico, Tommaso Trenti, Mario Luppi

**Affiliations:** 1 Department of Laboratory Medicine and Pathology, AUSL/AOU Policlinico, Modena, Italy; 2 Department of Medical and Surgical Sciences, University of Modena and Reggio Emilia, Hematology Unit, AOU Policlinico di Modena, Via Del Pozzo 71, 41124 Modena, Italy

Dear Editor,

Immunosuppressive therapy has emerged as promising therapeutic approach in the management of Coronavirus disease-19 (COVID-19) patients, who are often overwhelmed by dysfunctional immune responses [1]. However, some authors highlighted the risk related to unbalanced use of immunosuppressive treatments, since failure of antiviral immunity to control SARS-CoV-2 replication could underlie the hyper-inflammatory responses characterizing severe COVID-19 [2]. In critically ill COVID-19 patients, indeed, massive cytokine storms (including IL-6, TNF-α, and other inflammatory biomarkers), as well as increments of circulating neutrophils and monocyte activation, are typically observed together with low T lymphocyte counts and functional exhaustion of effector T cell responses [1, 3, 4]. Such ineffective and detrimental expansions of innate/humoral responses, alongside T cell suppression, are reminiscent of classical features of sepsis, which is currently defined as a life-threatening organ dysfunction induced by dysregulated host response to infection, being characterized not only by systemic hyperinflammation (SIRS) with related endothelial and organ damage, but also by impairment of adaptive T cell immunity. Moreover, the relevant coagulation disorders observed in end-stage COVID-19 could also well fit with the idea that severe COVID-19 possibly represents a peculiar clinicopathologic manifestation of viral sepsis.

To date, while clinical trials with immunosuppressive treatments (e.g., anti-IL-6 tocilizumab) are ongoing in COVID-19 patients [1], therapeutic approaches to enhance T cell functions have not yet been attempted in this setting. Importantly, immune checkpoint inhibitors (ICIs), such as anti-PD-1 and anti-PD-L1 monoclonal antibodies, originally developed to improve antineoplastic T cell immunity, are undergoing clinical investigation in septic patients [5]. Thus, it should be conceivable that, also in COVID-19 patients, ICIs may be tested to restore immune competence of exhausted T cell subsets and, in this context, to specifically improve the pivotal process of virus elimination, likely blunted in severe COVID-19. Of course, as for septic patients, the risk of immune-mediated complications (including inflammatory flares, pneumonitis, and systemic cytokine-release syndrome) could raise some concerns about the use of ICIs in COVID-19 patients. However, it should be noted that (i) autoimmune-like adverse events were not clinically evident in septic patients treated with ICIs [5] and (ii) tocilizumab represents a standard treatment for the management of such complications in cancer patients and could be promptly associated with ICIs in COVID-19 patients. While awaiting for the development of effective antivirals and vaccines against this life-threating coronavirus, we could harness the opportunity to try tuning patients’ immune system by using different immunomodulatory strategies now available, aiming to obtain more proper immune responses to SARS-CoV-2 infection and, hopefully, to reduce COVID-19-related mortality.

## Data Availability

Not applicable.
